# Generation of a Novel Transgenic Zebrafish for Studying Adipocyte Development and Metabolic Control

**DOI:** 10.3390/ijms22083994

**Published:** 2021-04-13

**Authors:** Yousheng Mao, Kwang-Heum Hong, Weifang Liao, Li Li, Seong-Jin Kim, Yinyi Xiong, In-Koo Nam, Seong-Kyu Choe, Seong-Ae Kwak

**Affiliations:** 1 Department of Medicine, Graduate School, Wonkwang University, Iksan 54538, Korea; maoyousheng1992@163.com (Y.M.); asdfdfghgh@naver.com (K.H.); 15070285697@163.com (W.L.); lilidaibaoli@163.com (L.L.); x1992889@gmail.com (Y.X.); 2 Department of Biomedical Science, Graduate School, Wonkwang University, Iksan 54538, Korea; mbunker1.21@gmail.com; 3 Institute of Brain Science, Wonkwang University, Iksan 54538, Korea; 8012645@hanmail.net; 4 Department of Microbiology, School of Medicine, Wonkwang University, Iksan 54538, Korea; 5 Institute of Wonkwang Medical Science, Wonkwang University, Iksan 54538, Korea; 6 Hanbang Cardio-Renal Research Center, Wonkwang University, Iksan 54538, Korea

**Keywords:** transgenic zebrafish, adipose tissue, live imaging, obesity

## Abstract

Zebrafish have become a popular animal model for studying various biological processes and human diseases. The metabolic pathways and players conserved among zebrafish and mammals facilitate the use of zebrafish to understand the pathological mechanisms underlying various metabolic disorders in humans. Adipocytes play an important role in metabolic homeostasis, and zebrafish adipocytes have been characterized. However, a versatile and reliable zebrafish model for long-term monitoring of adipose tissues has not been reported. In this study, we generated stable transgenic zebrafish expressing enhanced green fluorescent protein (EGFP) in adipocytes. The transgenic zebrafish harbored adipose tissues that could be detected using GFP fluorescence and the morphology of single adipocyte could be investigated in vivo. In addition, we demonstrated the applicability of this model to the long-term in vivo imaging of adipose tissue development and regulation based on nutrition. The transgenic zebrafish established in this study may serve as an excellent tool to advance the characterization of white adipose tissue in zebrafish, thereby aiding the development of therapeutic interventions to treat metabolic diseases in humans.

## 1. Introduction

The incidence of obesity and overweight individuals has been increasing worldwide over the past decades, and these two issues have attained a pandemic status [1]. Obesity can lead to the development of numerous health problems, including insulin resistance (IR), type 2 diabetes mellitus (T2DM), non-alcoholic fatty liver disease (NAFLD), cardiovascular disorders, and hypertension [2,3,4], and has, therefore, attracted considerable attention. In particular, visceral fat accumulation is considered as a major risk for the development of IR which then may facilitate the development of obesity-related metabolic syndrome [4,5]. In addition, reactive oxygen species seems to be a key player in the development of IR and the subsequent development of NAFLD or T2DM [4,6,7]. However, due to the complexity involving genetic, environmental, and behavioral effects to the development of obesity, the precise pathogenetic mechanisms leading to NAFLD and T2DM are not fully understood. Clinical evidence showed that some subjects with high degree of obesity can be considered metabolically healthy, suggesting that obesity per se may not always cause metabolic syndrome [8]. Moreover, limited adipose tissue expansion may also play a critical role in the development of T2DM and NAFLD [4,9]. Based on the adipose tissue expandability hypothesis, ectopic lipid load in non-adipocyte cells may induce persistent lipotoxicity such as IR, apoptosis, and inflammation which then forms the basis for the onset of T2DM and NAFLD. Therefore, integration of complex interactions between adipose tissues and other metabolic organs is pivotal to the understanding of energy balance and the development of obesity [10].

Since no animal model completely reflects biological pathways and diseases in humans, different model systems are utilized to compensate for intrinsic differences among them. Most of the current information regarding adipose tissue has been obtained using mammalian in vivo and in vitro models [9]. Zebrafish have attracted the attention of researchers because they provide an evolutionarily conserved and optically transparent system for developmental research [11]. In addition, zebrafish exhibit several advantages for metabolic research, including largely conserved physiology and anatomy compared with mammals, a broad range of genetic manipulations, and large-scale phenotypic screens [12]. The adipose tissues of zebrafish and mammals exhibit great similarities; first, human and zebrafish adipocytes contain large lipid droplets [13,14,15]. Second, they exhibit an energy storage function [15,16]. Third, human and zebrafish adipocytes exhibit a similar pattern of gene expression in terms of adipocyte differentiation and endocrine regulation [15,17,18]. Therefore, zebrafish have the potential to be used as an in vivo model to obtain new insights into the intrinsic mechanism of white adipose tissues, which may provide a new information of obesity and related comorbidity.

Based on the anatomical position, zebrafish adipose tissue can be divided into two major types; internal adipose tissue (IAT) and subcutaneous adipose tissue (SAT) [19]. IAT includes visceral and non-visceral adipose tissues such as paraosseal adipose tissue. SAT includes cranial (for example, ocular adipose tissue), truncal (for example, abdominal, dorsal, and ventral SAT) and appendicular SAT (for example, pectoral and caudal fin ray SAT). At present, in vivo observation of various zebrafish adipose tissues mainly involves the use of lipophilic dyes [20]. However, lipophilic dyes detect lipid droplets and do not necessarily reflect the size or morphology of the whole adipocyte. The accumulation of neutral lipids in some non-adipose tissues may interfere with data interpretation. Furthermore, it is not convenient or suitable for long-term imaging of adipose tissue using in vivo staining. Therefore, the development of a versatile and reliable zebrafish model for long-term monitoring of adipose tissues and adipocytes in vivo would greatly facilitate the examination of adipocyte dynamism to understand pathological progression of metabolic diseases in humans.

Phylogenetic analyses have revealed that fatty acid binding protein 11a (Fabp11a) and Fabp11b are homologous to mammalian fatty acid binding proteins, including FABP4, FABP5, FABP8, and FABP9 [21,22]. In situ hybridization validated that *fabp11a*, rather than *fabp11b*, continues to label adipocytes in major adipose depots [15]. These results suggest the selectivity of the *fabp11a* promoter to potentially be used to label adipocytes in vivo. In this study, we subcloned several different lengths of the *fabp11a* promoter region and performed adipocyte-specific transgenesis in zebrafish. We recovered a transgenic zebrafish containing the 1.5 kilobase-long proximal *fabp11a* promoter, which was sufficient to drive the expression of EGFP transgene, specifically in adipocytes throughout the adipose depots during development and in adult zebrafish. The transgenic zebrafish exhibited a reliable change in their adipogenic capacity depending on the nutritional status, suggesting a potential utility in live tracking of adipocytes under normal and pathogenic conditions.

## 2. Results

### 2.1. Proximal fabp11a Promoter Drives Adipocyte-Specific Expression of EGFP Transgene in Transgenic Animal

**Figure 1:**
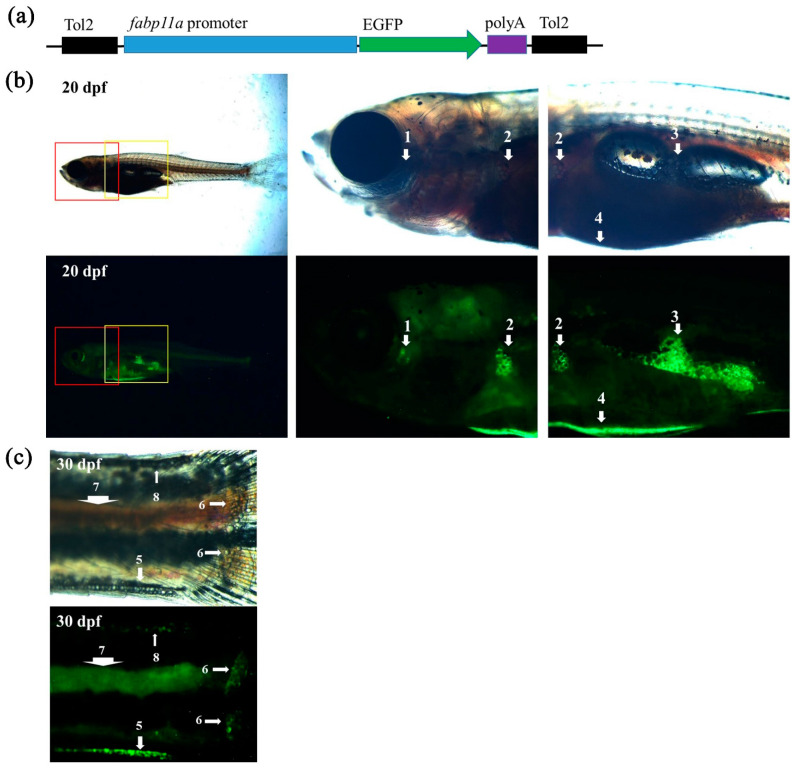
Visualization of adipose tissues in transgenic zebrafish. (**a**) Schematic drawing of the DNA construct used to generate the zebrafish transgenic line, Tg (*fabp11a*: EGFP). (**b**) Overview of a Tg (*fabp11a*: EGFP) at 20 dpf and zoom-in views of the boxed areas. Numbers denote adipose depots; 1, OCUAT (ocular adipose tissue); 2, PECSAT (pectoral fin subcutaneous adipose tissue); 3, VAT (visceral adipose tissue); and 4, ASAT (abdominal subcutaneous adipose tissue). (**c**) Imaging of a Tg (*fabp11a*: EGFP) zebrafish tail at 30 dpf. 5, VSAT (ventral subcutaneous adipose tissue); 6, CFRSAT (caudal fin ray subcutaneous adipose tissue); 7, POSAT (paraosseal adipose tissue); and 8, DSAT (dorsal subcutaneous adipose tissue).

Our approach to transgenesis using different lengths of the proximal *fabp11a* promoter generated F1 zebrafish that specifically expressed EGFP in adipocytes (Figure 1a). To further validate whether the EGFP signal in the stable transgenic line, we raised the next generation and examined the EGFP signal in various adipose tissues. To meet our needs, we found that the transgenic zebrafish line expressed EGFP in IAT, including visceral and non-visceral adipose tissues (Figure 1b,c). In addition, the transgenic zebrafish could also mark SAT, including cranial (such as ocular adipose tissue), truncal (such as abdominal, dorsal, and ventral SAT) and appendicular SAT (such as pectoral fin SAT and caudal fin ray SAT) (Figure 1b,c). The newly generated transgenic zebrafish line, Tg (*fabp11a*: EGFP), could hence reliably display various adipose tissues in vivo.

### 2.2. In Vivo Visualization of Adipocyte Morphology in Transgenic Zebrafish Larvae

**Figure 2:**
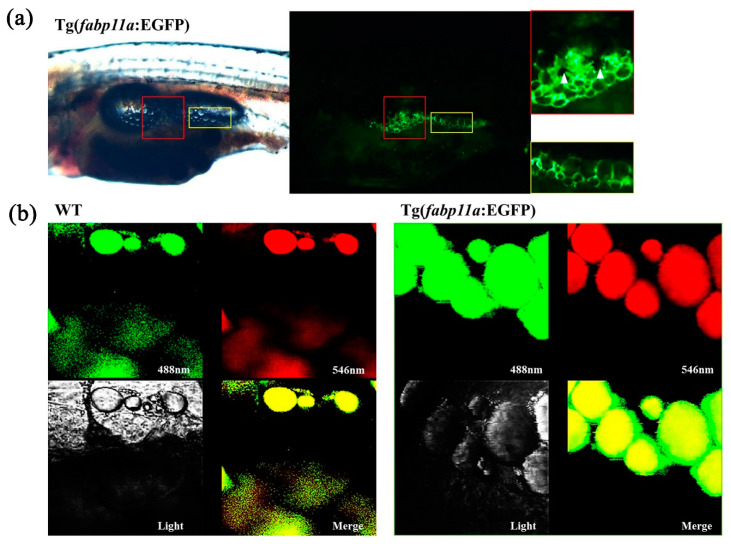
Visualization of adipocyte morphology in the transgenic zebrafish. (**a**) Images of trunk regions of zebrafish at 18 dpf and zoom-in views of the boxed areas. Red colored boxes show an area of pancreatic adipose tissue with multilayered adipocytes. Yellow colored boxes show an area of adipose tissue with single layer of adipocytes. White arrowheads indicate pigment cells. (**b**) Confocal images of adipocytes after Nile Red staining which is shown in both green and red. Green fluorescent signals are from both EGFP in the cytoplasm and Nile Red-stained lipid droplet, whereas red fluorescent signal is only from lipid droplet. Merged images show yellow lipid droplet and green cytoplasm.

We examined the adipocyte morphology in vivo using the transgenic zebrafish line, Tg (*fabp11a*: EGFP) (Figure 2a). A typical adipocyte morphology is a single, large lipid droplet with a small nucleus; the adipocytes detected through the expression of EGFP in our transgenic zebrafish contained a large, black center with surrounding area in green (Figure 2a). We presumed that the black center was the lipid droplet, and the green periphery was the cytosol containing EGFP. This was confirmed by Nile Red staining. Nile Red marked the lipid droplets in the adipocytes of wild type (WT) and transgenic zebrafish, whereas the expression of EGFP was broadly detected in the cytosol of the adipocytes of the transgenic zebrafish, differentiating the transgenic zebrafish from their WT siblings (Figure 2b). Moreover, we were able to detect the morphology of a single adipocyte in vivo using the transgenic zebrafish line (Figure 2b).

### 2.3. In Vivo Monitoring of Transgenic Zebrafish Larvae Revealed Asymmetrical Development of Visceral Adipose Tissues 

**Figure 3:**
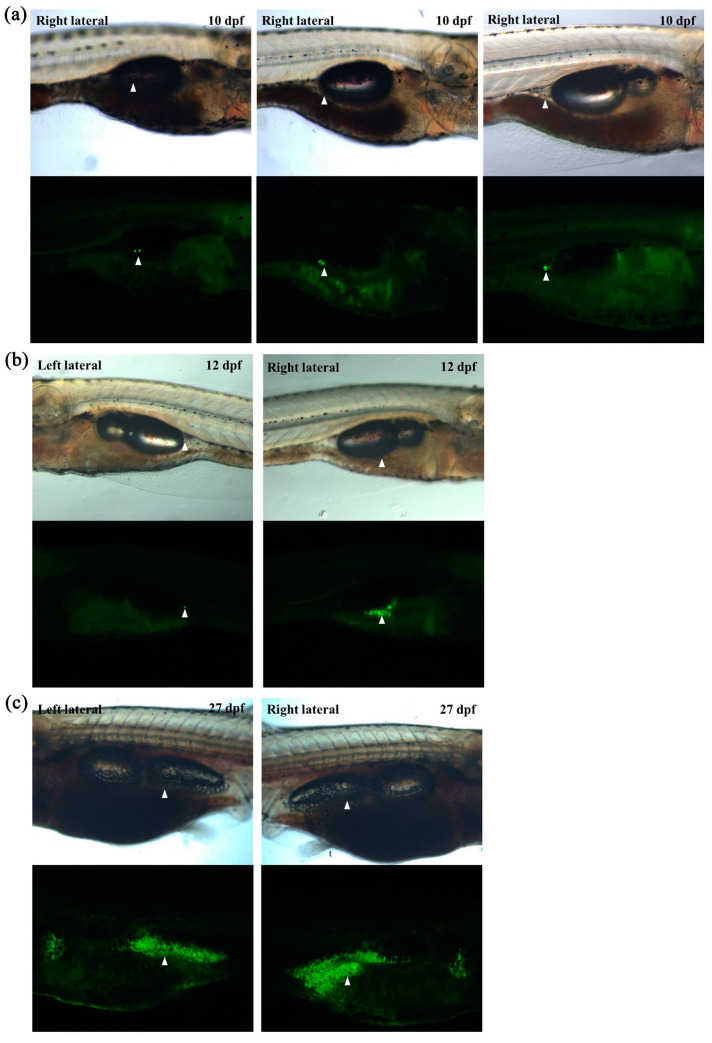
In vivo monitoring of visceral adipose tissue development in larvae using transgenic zebrafish. (**a**) Representative images of the first adipocyte emerging in three independent larvae at 10 dpf. (**b**) Representative images of visceral adipose tissues at 12 dpf. The first adipocyte of left lateral emerged. (**c**) Representative images of visceral adipose tissues at 27 dpf. White arrowheads indicate visceral adipose tissue.

As our newly generated transgenic zebrafish could be used to detect adipose tissues as well as a single adipocyte, we decided to monitor adipose tissue development in vivo. After monitoring at least 50 Tg (*fabp11a*: EGFP) larvae continuously from 5 to 30 dpf, we confirmed a previously reported finding on the development of adipose tissues in zebrafish; the earliest emerging adipocyte is a visceral adipocyte that appears adjacent to the pancreas in a relatively constant position at approximately 9–10 dpf (Figure 3a). In addition, we found that the development and distribution of visceral adipose tissues in zebrafish are asymmetrical; right lateral visceral adipose tissue emerges at least 2 days earlier (Figure 3a,b) and grows much faster than left lateral one (Figure 3b,c). These results clearly suggested that the newly generated transgenic zebrafish line may be useful for continuously monitoring adipose tissue development and may potentially enable to investigate adipocyte hypertrophy and hyperplasia in vivo.

### 2.4. Transgenic Zebrafish Could Be Used to Monitor Nutrition-Dependent Adipocyte Development and Maintenance In Vivo

**Figure 4:**
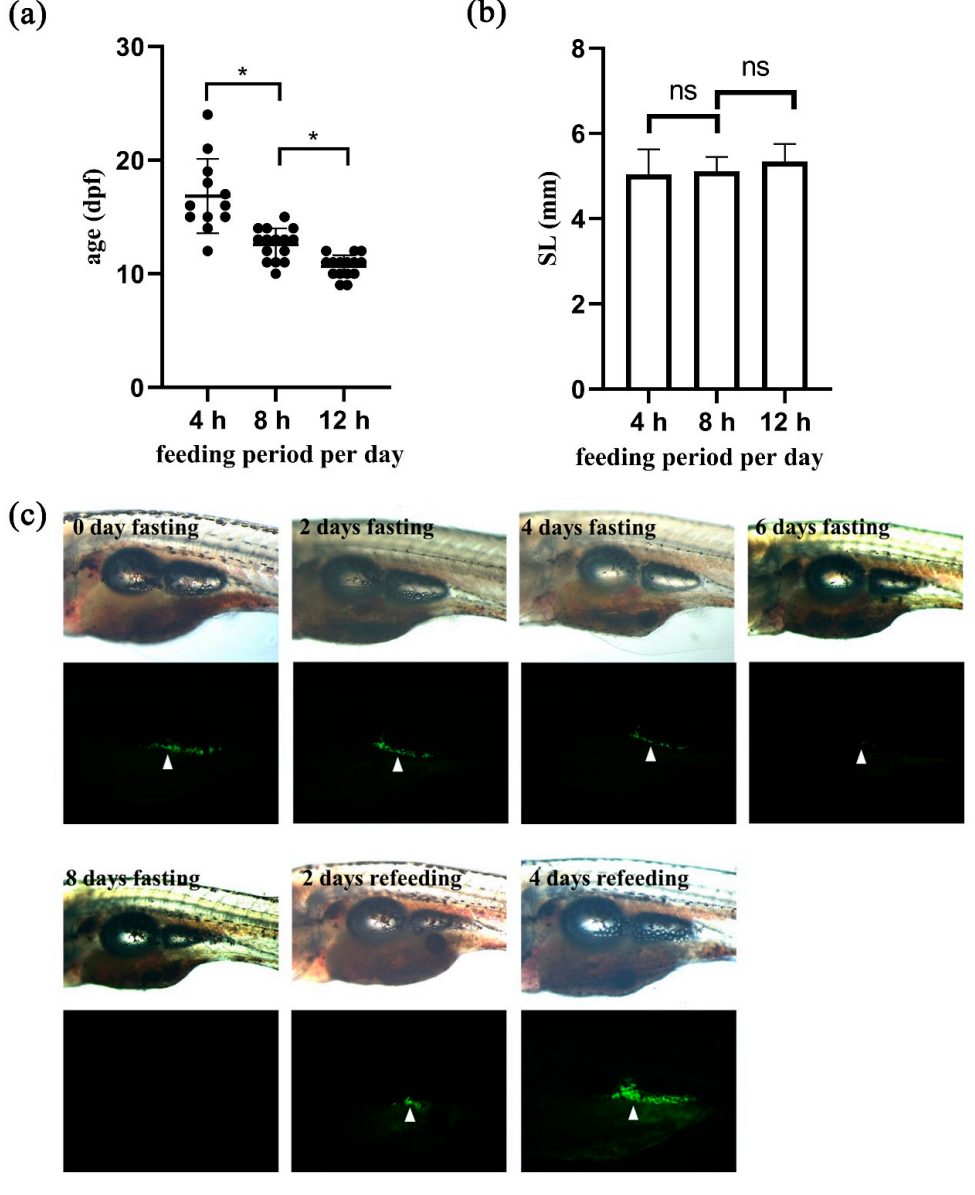
Nutrition influences on adipocyte development and maintenance. (**a**,**b**). Zebrafish embryos at 5 dpf were fed for different durations per day as indicated, and adipocyte development was examined. *n* ≥ 12. Repeat = 3. * indicates *p* < 0.05, and ns indicates *p* > 0.05. (**a**) Graphical presentation of the age of larvae that developed the first adipocyte in three groups. (**b**) Graphical presentation of the body length of larvae that developed the first adipocyte in three groups. ns, not significant. (**c**) 16 dpf larvae were selected and subjected to fasting for 8 days, and then refed for another 4 days. Representative bright field and fluorescent images are shown to orient the larvae with visceral adipose tissues marked by white arrowheads. *n* ≥ 5. Repeat = 3.

To verify the utility of the newly generated transgenic zebrafish in live imaging, we examined the relationship between nutrition and adipocyte development. First, we tested whether food supply influenced on the appearance of the first adipocyte relative to body growth. We applied different feeding regimes and found that the group of zebrafish that received 12 h-feeding per day developed the first adipocyte as early as 9 dpf and at 10.7 dpf on average (Figure 4a). On the other hand, the group that was subjected to a 4 h- feeding regime developed the first adipocyte at 16.8 dpf on average (Figure 4a), suggesting that adipocyte development may be dependent on nutrition. Interestingly, although zebrafish belonging to different groups showed differential growth rates, the average body length of zebrafish when their first adipocytes were detected was not different (*p* > 0.05, Figure 4b). These results indicate that body length is the determining factor for the appearance of the first adipocyte, whereas nutrition regulates the growth rate during zebrafish development.

We further investigated the relationship between nutrition and adipocyte maintenance through live imaging of transgenic Tg (*fabp11a*: EGFP) zebrafish. We found that visceral adipose tissues gradually disappeared after withdrawal of food supply and reappeared after refeeding (Figure 4c). In particular, we found that adipose tissues were essentially undetectable after 8 days of fasting, and that they were reestablished within 2 days after refeeding (Figure 4c). Notably, starvation blocked adipocyte development in zebrafish, as we did not find any newly emerging adipocytes (Figure 4c).

## 3. Discussion

In this study, we generated transgenic zebrafish, Tg (*fabp11a*: EGFP), to visualize adipose tissues and adipocytes in vivo. We understand that there is currently no zebrafish model that specifically labels adipocytes in vivo; therefore, the newly generated transgenic zebrafish have tremendous potential for use in adipocyte research. We found that the transgenic zebrafish could detect all types of adipose tissues during adipocyte development and after maturity. In addition, we were able to detect the morphology of single adipocytes of the transgenic zebrafish; hence, this transgenic zebrafish line can be potentially used to identify the origin of adipocytes during adipocyte hyperplasia and studying the size, lipid accumulation, numbers, and transformative potential of adipocytes during the life cycle of the zebrafish.

Although one needs to be careful to interpret data obtained from an animal model due to its innate difference from human, we validated that the transgenic zebrafish were well suited for monitoring the development of adipose tissues in vivo. According to our observations, the first adipocyte emerged near the pancreatic area when the zebrafish body length exceeded 5 mm. Interestingly, we found that the visceral adipose tissues were asymmetrically developed, which necessitated imaging of both the left and right sides of the zebrafish when adipose tissues were examined. We also validated that the transgenic zebrafish were well suited for monitoring quantitative changes in adipose tissues in vivo. In particular, a fasting-refeeding experiment using the transgenic zebrafish confirmed their utility in monitoring adipocyte development and maintenance based on the nutrition status. The utility of our transgenic zebrafish could be augmented when combined with transparent transgenic zebrafish [23]; adipose tissue development according to different nutrition status could be analyzed throughout the life cycle of zebrafish to study causal relationship between obesity and the onset and progression of NAFLD. Moreover, a seemingly paradoxical “metabolically healthy obese” [4] could also be addressed by examining inter-relationship between adipose tissues and other metabolic organs, such as liver and muscle.

In summary, transgenic zebrafish Tg (*fabp11a*: EGFP) serve as a versatile and reliable model for the long-term monitoring of adipose tissue and adipocytes in vivo. Considering the capability of a library screen combined with the power of genetics that the zebrafish model can provide, our transgenic zebrafish may be useful to advance obesity-related research and preventative medicine.

## 4. Materials and Methods

### 4.1. Zebrafish Husbandry and Experimental Conditions

Zebrafish were raised and maintained under a 14 h light/10 h dark cycle using standard protocols [24]. The embryos were collected, developed in egg water (5 mM NaCl, 0.17 mM KCl, 0.33 mM CaCl_2_, 0.33 mM MgSO_4_) and staged according to standard protocols [25]. For the adipocyte development experiment, larvae at 5 days post fertilization (dpf) were randomly divided into three groups. The three groups were fed commercial food and fresh artemia for 4, 8 and 12 h, respectively, and the surplus food was removed after feeding by changing the water. For the fasting and refeeding experiment, 16 dpf larvae were subjected to fasting for 8 days and were then fed for 4 days. All the larvae were raised in a transparent 2 L tank under a 14 h light/10 h dark cycle, with less than 15 larvae per tank. Each experiment was performed at least twice.

### 4.2. Generation of DNA Constructs and Transgenic Lines

The 1.5 kilobase-long proximal *fabp11a* promoter region was amplified from total genomic DNA extracted from zebrafish embryos at 24 hpf using a standard PCR protocol and cloned into the pCRII-Topo vector (Invitrogen, Carlsbad, CA, USA). The primer sequences used for PCR were as follows: forward primer 5′-AAGGATCCCCTGGCTTGCAGTTTATAGAAGCAG-3′ and reverse primer 5′-CGACCGGTTTTCCCGAATAATGATGCTCCTCT-3′. The *fabp11a* promoter was excised from the pCRII-Topo-*fabp11a* plasmid using BamHI (NEB, Ipswich, MA, USA) and sub-cloned into the BamHI site in the Mini-Tol2-EGFP plasmid. Purified mini-Tol2-*fabp11a*: EGFP plasmid (25 ng) and in vitro synthesized Tol2 *transposase* mRNA (50 ng) was microinjected into embryos at the 1-cell stage. All the injected embryos were raised and mated with wild type (WT) zebrafish to generate F1 zebrafish. F1 zebrafish with green fluorescence were observed and collected at various larval stages using a Leica M165FC microscope. F2 and F3 zebrafish were also generated and imaged in this study.

### 4.3. Nile Red Staining and Imaging

In vivo Nile Red staining was performed as previously described [26]. Briefly, Nile Red (Invitrogen, Carlsbad, CA, USA) was diluted to a final working concentration of 0.5 μg/mL egg water and then used for staining in the dark for 30 min. At the end of staining, larvae were anesthetized with 0.03% tricaine (Sigma-Aldrich, St. Louis, MO, USA), mounted in 3% methylcellulose, and imaged using a confocal microscope (Olympus IX81, Fluoview FV1000).

### 4.4. In Vivo Imaging and Body Length Measurement

Zebrafish larvae were anesthetized with 0.05% tricaine (Sigma-Aldrich, St. Louis, MO, USA) in egg water and then mounted in 3% methylcellulose for imaging. Imaging was performed using a Leica M165FC microscope, equipped with 488 nm laser. After imaging, the zebrafish were released into fresh water and recovered quickly. The distance between the tip of the snout and the caudal peduncle was measured and used as the larval standard length (SL) using ImageJ software (NIH, Bethesda, MD, USA).

### 4.5. Statistical Analyses

All values are reported as mean ± standard errors of the mean (SEM). The Student’s unpaired t-test in GraphPad Prism 8 software (GraphPad, San Diego, CA, USA) was used to determine statistical significance. Significance was set at *p* < 0.05.

## Data Availability

Not applicable.
